# A response‐locking protocol to boost sensitivity for fMRI‐based neurochronometry

**DOI:** 10.1002/hbm.25026

**Published:** 2020-05-09

**Authors:** Shivakumar Viswanathan, Rouhollah O. Abdollahi, Bin A. Wang, Christian Grefkes, Gereon R. Fink, Silvia Daun

**Affiliations:** ^1^ Cognitive Neuroscience Institute of Neuroscience and Medicine (INM‐3), Research Centre Jülich Jülich Germany; ^2^ Medical Faculty, University of Cologne & Department of Neurology University Hospital Cologne Cologne Germany; ^3^ Institute of Zoology, University of Cologne Cologne Germany

**Keywords:** cognition, decision making, fMRI, movement, neurophysiology, reaction time

## Abstract

The timeline of brain‐wide neural activity relative to a behavioral event is crucial when decoding the neural implementation of a cognitive process. Yet, fMRI assesses neural processes indirectly via delayed and regionally variable hemodynamics. This method‐inherent temporal distortion impacts the interpretation of behavior‐linked neural timing. Here we describe a novel behavioral protocol that aims at disentangling the BOLD dynamics of the pre‐ and post‐response periods in response time tasks. We tested this response‐locking protocol in a perceptual decision‐making (random dot) task. Increasing perceptual difficulty produced expected activity increases over a broad network involving the lateral/medial prefrontal cortex and the anterior insula. However, response‐locking revealed a previously unreported functional dissociation within this network. preSMA and anterior premotor cortex (prePMV) showed post‐response activity modulations while anterior insula and anterior cingulate cortex did not. Furthermore, post‐response BOLD activity at preSMA showed a modulation in timing but not amplitude while this pattern was reversed at prePMV. These timeline dissociations with response‐locking thus revealed three functionally distinct sub‐networks in what was seemingly one shared distributed network modulated by perceptual difficulty. These findings suggest that our novel response‐locked protocol could boost the timing‐related sensitivity of fMRI.

## 1 INTRODUCTION

During a cognitive process, the timeline of *when* neural populations become active relative to each other and to behavioral events is crucial to elucidate how that process is implemented at the neural level (Calhoun, Miller, Pearlson, & Adali, 2014; Elia Formisano & Goebel, 2003; King & Dehaene, 2014; Kutas, McCarthy, & Donchin, 1977; Posner, 2005). Identifying this timeline with functional Magnetic Resonance Imaging (fMRI) faces the constraint that neural dynamics on the millisecond timescale are measured only indirectly via comparatively slow multi‐second changes in regional blood flow and oxygenation (Arthurs & Boniface, 2002; Ekstrom, 2010; Kim & Ogawa, 2012; Logothetis, Pauls, Augath, Trinath, & Oeltermann, 2001; Ogawa, Menon, Kim, & Ugurbil, 1998). Due to the ensuing temporal distortions, the timing structure of a cognitive process is a critical determinant of whether its timeline can be identified with fMRI (E. Formisano & Goebel, 2003; R. S. Menon, Luknowsky, & Gati, 1998; R. S. Menon & Kim, 1999). Therefore, optimizing experimental paradigms to enhance sensitivity for fMRI‐based neurochronometry is warranted.

Since fMRI distorts the timescale, one strategy to increase chronometric sensitivity is to modify a paradigm's timescale to be more compatible with that of the Blood‐Oxygen‐Level‐Dependent (BOLD) signal (E. Formisano et al., 2002; Gratton et al., 2017; Ploran et al., 2007; Ploran, Tremel, Nelson, & Wheeler, 2011; Richter et al., 2000). However, fMRI's timescale distortion poses a problem illustrated by the following scenario. Suppose *A* and *B* are neurocomputational stages of a cognitive task, where *A* always precedes *B*. Interpreting *A*'s functional relationship to *B* depends on their timing relative to the behavioral events of the task. If both *A* and *B* always occur between the stimulus onset and the behavioral response, then *A*'s relationship to *B* would be interpreted differently than in a scenario where *A* always precedes the behavioral response while *B* follows it. To avoid such ambiguities, one solution is to “time‐lock” neural dynamics to behavioral events by directly integrating behavioral events into the neural timeline, as is done in electroencephalography (EEG) and magnetoencephalography (MEG). This solution is, however, unavailable to fMRI due to the differing timescales of the behavioral events (milliseconds) and the BOLD signal (seconds). To address this issue, we investigated a novel time‐locking strategy based on the paradigm's temporal structure. Rather than modifying a paradigm's overall timescale, the timing of behavioral events on each trial was used to modify that trial's timescale *selectively*.

The proposed strategy is directed toward typical experimental paradigms where the reference time‐point is the stimulus onset when the brain receives an external information input, and the ensuing action (e.g., pressing a button) represents the output of the cognitive process. On this stimulus‐referenced timeline, the input‐to‐output transformation is attributable to the neural processes operating in the time interval between the stimulus and the response, that is, the response time (RT) (Donders, 1868; Luce, 1991; Sternberg, 1969). Identifying these time‐bound neural processes, however, requires them to be accurately distinguished from processes operating *outside* the RT interval, namely, in the pre‐stimulus and post‐response periods. This within/outside distinction is the target of the proposed strategy. Unlike pre‐stimulus neural dynamics (cf. Busch, Dubois, & VanRullen, 2009), the post‐response neural dynamics are RT‐irrelevant as they can no longer influence the RT. Furthermore, the pre‐response and post‐response periods are defined relative to the response event which is independent of the stimulus. Based on this rationale, we devised a response protocol to modulate the duration of the post‐response processes in a response‐locked manner. We conjectured that this approach enhances the distinguishability of BOLD signal dynamics with a neural origin in the post‐response (RT‐irrelevant) versus pre‐response (RT‐relevant) periods.

**FIGURE 1:**
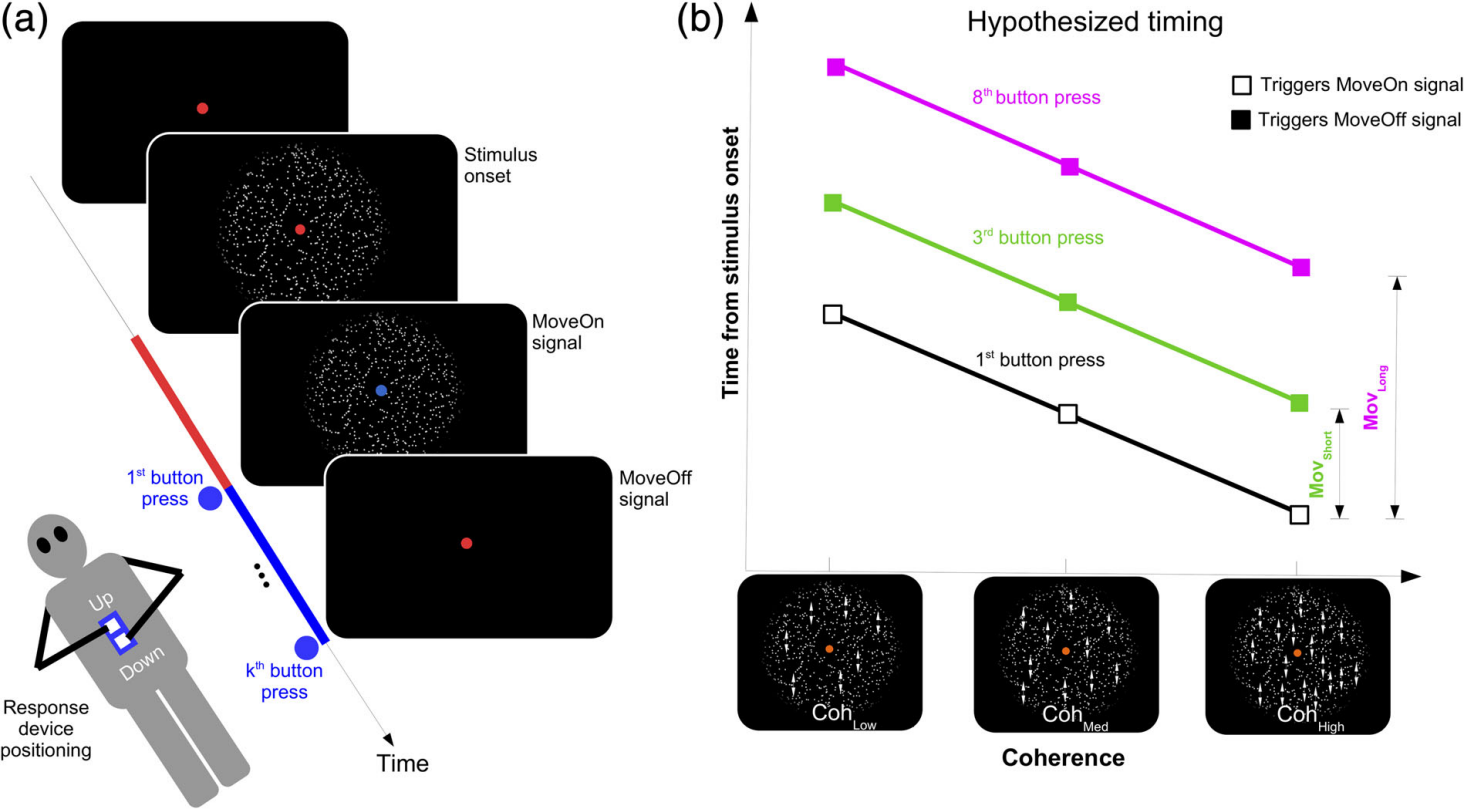
Experimental paradigm. (a) Trial schematic. Each trial started with the stimulus onset and ended with the stimulus offset. Participants lay supine in the scanner, and the response device was positioned vertically on their midline. The colors of the central fixation point and their relative durations are depicted using thick lines on the time‐axis. The fixation point was colored red at stimulus onset with a change to blue following the first button press (i.e., MoveOn signal) and changed back to red after either *k* = 3 or *k* = 8 button‐presses (i.e., MoveOff signal). (b) The hypothesized timing between the different stimuli is shown. The MoveOff signal was displayed after either three or eight button presses (filled squares). The timing of the first button press (open squares) varies with stimulus coherence (black line). The coherently moving dots are illustrated here with arrows and were not displayed in the experiment. The interval between the first button press to the third/eighth button press was assumed to be unaffected by stimulus coherence

To test this conjecture, the response protocol was embedded within a perceptual decision‐making task (Donner, Siegel, Fries, & Engel, 2009; Gold & Shadlen, 2007; H. R. Heekeren, Marrett, Ruff, Bandettini, & Ungerleider, 2006; T. Liu & Pleskac, 2011; Tosoni, Galati, Romani, & Corbetta, 2008). On each trial (Figure 1a), participants viewed a random moving dot stimulus and reported its motion direction by pressing a corresponding button. Rather than a single button‐press, the selected button had to be pressed repetitively and rapidly until a halt was signaled with a MoveOff stimulus. Importantly, the timing of this MoveOff stimulus was *response‐locked*, that is, defined relative to the first button‐press on that trial.

Each trial's duration was the sum of two intervals: (a) from the stimulus to the first button press (i.e., the RT) and (b) from the first button‐press until the MoveOff stimulus (i.e., the movement time [MvT]). The RT and MvT were independently varied in an RT × MvT design by modulating the stimulus noise levels and the response‐locked timing of the MoveOff stimulus (Figure 1b). With this design, the mean BOLD differences between trial‐types with the same mean RT but different mean MvTs should have a response‐locked neural origin, namely, *after* the first button‐press.

We evaluated whether these predicted response‐locked differences in the fMRI data increased sensitivity in the context of the perceptual decision‐making task. Even though motion direction is a property of the visual stimulus, stimulus‐related RT‐modulations have surprisingly been found to modulate action‐related neural processes in non‐human primates but this modulation in the human brain has been controversial (Donner et al., 2009; Filimon, Philiastides, Nelson, Kloosterman, & Heekeren, 2013; Gold & Shadlen, 2007; H. R. Heekeren et al., 2006; H. R. Heekeren, Marrett, & Ungerleider, 2008; Resulaj, Kiani, Wolpert, & Shadlen, 2009; Tosoni et al., 2014; Tosoni et al., 2008). We hypothesized that activity modulation by RT *and* MvT would provide a strict functional (rather than anatomical) criterion to identify stimulus‐modulated regions (pre‐response) with a measurable role in action execution (post‐response).

## 2 MATERIALS AND METHODS

### 2.1 Participants

Thirty‐three healthy, young volunteers (mean age: 25.6 years [*SD* = 2.8 years], range: 19–32 years, female = 16) participated in the experiment and received financial compensation. Participants were right‐handed (mean score = 81.2% [*SD* = 18.2%] [Edinburgh Handedness Inventory (Oldfield, 1971)]), had normal or corrected‐to‐normal vision, no history of psychiatric or neurological diseases, and no contraindications for MRI scanning. Participants were additionally prescreened for their ability to perform the task. The local ethics committee approved the study, which complied with the Declaration of Helsinki. All volunteers provided their informed consent before the experiment.

Statistical analyses reported here are based on datasets from 30 (of the 33) participants due to the exclusion of 3 datasets based on quality considerations (see details below).

### 2.2 Stimulus specification

Visual stimuli were generated and displayed using the Presentation® Software (Neurobehavioral Systems, Inc.) on an LCD screen (size: 68.6 cm [diagonal], resolution: 1,200 pixels × 800 pixels, frame rate: 60 Hz). The screen was located behind the scanner and was viewed via a mirror installed on the head coil.

The stimulus on each trial was a centrally displayed random dot kinematogram (RDK) (Morgan & Ward, 1980; Williams & Sekuler, 1984) (Figure 1a). The RDK consisted of 600 white‐colored dots (diameter: 0.11**°** visual angle [v.a.], speed: 8**°** v.a./s) moving within an invisible circle (diameter: 6**°** v.a., background‐color: black). The dots were distributed equally in each of the circle's quadrants. Each dot had a finite life that ended either (a) after 0.5 s (30 frames), or (b) when the dot moved outside the invisible circle's periphery. A dot that met either criterion was replaced on the next frame by a new dot that appeared at a random location within the circle. To reduce the abruptness with which dots disappeared at the circle's periphery, the circle was windowed by a Gaussian luminance envelope so that dots appeared to be brighter at the circle's center and progressively dimmer toward the periphery.

On every frame, each dot was assigned a motion direction based on two trial‐specific parameters: (a) coherence (Coh) and (b) global direction (*D*). The parameter Coh specified the proportion of dots to be assigned to the (coherent) motion direction *D*. These dots with a coherent motion direction were selected randomly on each frame while the remaining dots were each assigned a direction selected randomly over the uniform range [0°, 359°]. For example, in an RDK with *D =* 90° and Coh = 60%, 360 randomly selected dots (60% of 600) on each frame would be assigned a motion direction of 90° while each of the remaining 240 dots would be assigned a random motion direction. Due to this randomization procedure, a dot with a direction *D* in one frame would likely have a different direction in the next frame and vice versa, thus making it difficult to infer the direction *D* by tracking the motion of any single dot.

The global motion direction *D* could take either of two values: 90° (i.e., upward) or 270° (i.e., downward). A random jitter was added to the value of *D* on each trial to limit habituation and learning effects from repeated exposure to the same motion directions (K Ball & Sekuler, 1982; Karlene Ball & Sekuler, 1987). This jitter was selected from a discontinuous range ([−30°, −10°], [+10°, +30°]) to minimize motion along the cardinal axes. The coherence Coh had three levels {Coh_High_, Coh_Med_, Coh_Low_} that were calibrated per participant to produce mean response times (i.e., time to report an RDK's direction) of 600 ms, 750 ms, and 900 ms. These RT values were selected to evoke neural activity differences that can be detected with fMRI and EEG (Philiastides & Sajda, 2007; Yarkoni, Barch, Gray, Conturo, & Braver, 2009). The mean coherence values across participants were Coh_High_ = 31.2% (*SD*: 8.7%), Coh_Med_ = 18.4% (*SD*: 5.7%), and Coh_Low_ = 12.7% (*SD*: 4.2%).

Finally, a small static disc (diameter: 0.2° v.a.) was centrally displayed over the whole experiment to serve as a fixation point and as a cue at different stages of the experiment based on its color (red, blue, or gray).

### 2.3 Paradigm

A crucial context for the experimental paradigm was the response setup. Response events in the experiment were index finger button presses, which were recorded with an MRI compatible LUMItouch response pad (Photon Control Inc., Burnaby, BC, Canada). The response device was placed on the participant's chest to align the buttons vertically along the participant's midline (Figure 1a). Two buttons were designated as the upper (closer to the participants' head) and lower (closer to their feet) buttons. Participants positioned their hands to press the upper and lower button with their left and right index finger, respectively. The assignment of left/right index fingers to press the upper/lower buttons was counterbalanced across participants, that is, half the participants pressed the upper button with the right hand while the other half used the left hand.

The up/down orientation for both index finger positions and RDK motion directions ensured that the response rather than the stimulus primarily drove any lateralization of brain activity. If the stimuli/responses instead had a left/right orientation (as in Heekeren et al., 2006; de Lange, Rahnev, Donner, & Lau, 2013; Kelly & O'Connell, 2013) then lateralized neural activity linked to both the RDK's motion direction (for example, due to attention orienting) and the corresponding response (i.e., with the left index finger) would be lateralized to the same (i.e., right) hemisphere.

Figure 1a is a schematic of each trial's organization and timing. Each trial began with the display of the RDK stimulus with a red‐colored fixation point at its center. Participants judged whether the moving dots of the RDK had an upward or downward direction and reported this perceptual decision by pressing the corresponding upper or lower button repeatedly and as rapidly as possible. The first button‐press of the response triggered a change in the fixation point's color from red to blue. Following this color change, participants now had to monitor the fixation point's color to detect a second color change while repetitively pressing the selected button. The second color change (from blue to red) was the signal to halt the response immediately and marked the end of the trial. For clarity, we henceforth refer to these two color changes as the MoveOn (red to blue) and MoveOff (blue to red) signals. The RDK was continuously displayed over the trial's entire duration.

Unknown to the participants, the interval between the first button‐press and the halt‐signal (i.e., the blue‐to‐red color change) was controlled by their behavior. The button presses were counted in real‐time as they were produced, and the MoveOff signal was displayed when this real‐time count reached a pre‐defined target value. This target value was either 3 or 8 button presses corresponding to a “short and “long” movement duration (denoted as Mov_Short_ and Mov_Long_).

Real‐time counting was used rather than a pre‐defined period so that the number of movements associated with Mov_Short_ and Mov_Long_ was consistent across individuals to equate for interindividual differences in maximum tapping rate (cf., the clinical Finger‐Tapping Test: Shimoyama, Ninchoji, & Uemara, 1990). To ensure that the time to complete three button presses was shorter than to complete eight button presses, a missed trial occurred if neither button was pressed within 1,800 ms following stimulus onset or if the required number of button presses was not completed within 1 s (for Mov_Short_) or 2.5 s (for Mov_Long_). The MoveOff signal was presented while participants were pressing a button rapidly and repeatedly. Consequently, we assumed that instructing a halt would lead to additional button presses before all movements ceased. The target number of button‐presses 3 and 8 were selected so that the relative time differences would be maintained even with a few excess button presses.

To prevent the perceptual decision from being prioritized over the response demands, the RDK stimulus was continuously displayed until the MoveOff signal to de‐emphasize the distinction between the RT and MvT intervals. This continuous stimulus display also avoided a confound between (a) the neural processes associated with the response onset and (b) processes evoked by the stimulus offset (also see Kelly & O'Connell, 2013). Strictly speaking, the MoveOff signal was redundant information as it coincided with the RDK's disappearance from the screen but this redundant color cueing served to emphasize that the response requirements were a crucial part of the task.

### 2.4 Procedure and training

Participants attended two sessions on separate days: instruction and training outside the scanner (Session 1), and the main experiment in the scanner (Session 2).

Training began with detailed task instructions delivered verbally, following a script. Next, participants were familiarized with the RDK by performing the task on stimuli that steadily increased in difficulty, going from 100% coherence to 20% in stepwise reductions of 20% every 20 trials. This procedure was repeated with additional instruction as needed until an overall accuracy of 90% was reached. After familiarization, a calibration procedure followed to identify three coherence values {Coh_High_, Coh_Med_, Coh_Low_} that would produce mean RTs of 600 ms, 750 ms, and 900 ms, respectively. Briefly, calibration started with an initial coherence estimate for each target RT (<Coherence, RT>: <90%, 600 ms>; <80%, 750 ms>; <70%, 900 ms>). This initial estimate was then iteratively refined based on the participant's performance using the Parametric Estimation by Sequential Testing (PEST) algorithm (Lieberman and Pentland, 1982). The iterative coherence adjustments (step size: maximum = 8%, minimum = 0.5%; maximum trials: 50) continued until each target RT was reached with an accuracy of 90%. The minimum possible coherence value was set to 7% (based on our pilot studies). Calibration for all target RTs was performed concurrently by interleaving trials for each target. For robustness, this calibration procedure was repeated three times, and the mean coherence across these repetitions was used as the final calibrated value. Participants were unaware that these calibrated coherence values would determine the stimuli that they would be exposed to in the main experiment. Finally, participants performed a shortened version of the main experiment to confirm whether the calibrated coherence values produced the expected RT ordering in the actual experimental scenario. If the mean RT was lowest for Coh_High_, highest for Coh_Low_, and intermediate for Coh_Med_ (with a mean accuracy ≥ 85% per coherence), then calibration was deemed successful, and participants were invited to the main experiment.

In the second session, participants were re‐instructed and practiced the task before entering the scanner to perform the main experiment. The assignment of left/right index fingers to press the upper/lower buttons was the same for both sessions. Special precautions were taken to firmly secure participant's elbows in the scanner to minimize head movements while responding. Due to the long duration of the experiment (~96 min), participants received rest periods of several minutes between runs when scanning was halted.

### 2.5 Trial design

Trials were defined by three independent factors: RDK direction {Up, Down} × Coherence {Coh_High_, Coh_Med_, Coh_Low_} × Mov {Mov_Short_, Mov_Long_}. The experiment had a total of 624 trials with 52 trials in each of the 2 × 3 × 2 = 12 conditions.

Trials were organized into four runs (~24 min each) in a rapid‐event design, and each run started and ended with an 11 s task‐free period to minimize transient effects of the starting and ending of the session on task‐related BOLD activity. A run was divided into four task‐blocks (39 trials each) interleaved with 12 s task‐free periods that were indicated by a gray‐colored fixation point. Each task‐block ended with a feedback screen (3 s) displaying the numerical accuracy (in percent) and the number of missed stimuli on that block. The inter‐trial interval varied from 4 to 8 s. To de‐confound task and scanner timings, the trial onset was jittered relative to the start of a new EPI (uniformly, randomly in the range [0 ms, 2,200 ms (=1TR)] discretized into 100 ms intervals). To improve randomization (T. T. Liu, Frank, Wong, & Buxton, 2001; Thomas T. Liu & Frank, 2004), three null trials (duration: 1800 ms) were included in each block. All trials were displayed in a pseudorandomized order specified by a Maximum Length Sequence (or m‐sequence) (Aguirre, 2007; Aguirre, Mattar, & Magis‐Weinberg, 2011; Buračas & Boynton, 2002) that ensured a counterbalanced presentation of the 13 trial types (12 conditions +1 null event).

Since the stimulus–response mapping differed between participants (see Paradigm), before statistical modeling, each trial was recategorized based on whether that trial required a response with the left or right index finger (rather than the RDK direction) due to our focus on action‐related processes. With this re‐categorization, the factors defining the 12 conditions of interest were: Hand {Left, Right} × Coh {Coh_High_, Coh_Med_, Coh_Low_} × Mov {Mov_Short_, Mov_Long_}.

### 2.6 fMRI data acquisition and preprocessing

Functional and structural MR images were acquired on a 3T MR scanner (Siemens Tim Trio, Erlangen, Germany) using a circularly polarized (CP) head coil, as part of a simultaneous fMRI‐EEG study. Image preprocessing and statistical analysis were performed with SPM12 (version 6,685) software (Wellcome Centre for Human Neuroimaging, London, UK) within MATLAB 8.3 R2014a (MathWorks Inc., Natick, MA) on a Linux operating system (core version 3.16.0‐5‐amd64, Debian 8.10 Jessie).

Functional images were measured using a T2*‐weighted gradient‐echo planar imaging (EPI) sequence (repetition time (TR): 2200 ms, echo time (TE): 30 ms, flip angle (FA): 90**°**, field of view (FoV): 200 mm × 200 mm). Each volume had 36 slices (ascending series, thickness: 3.0 mm, inter‐slice gap: 0.47 mm) with an in‐plane resolution of 3.1 mm × 3.1 mm (matrix size: 64 × 64). Additionally, an anatomical scan was obtained per participant with a T1‐weighted magnetization‐prepared rapid gradient echo (MPRAGE) sequence (TR: 2,250 ms, TE: 3.03 ms, FA: 9**°**, TI: 900 ms, FOV: 256 mm × 245 mm) to obtain a high‐resolution structural image (176 slices, matrix size: 256 × 256, inter‐slice gap: 0.5 mm, voxel size: 1.0 mm × 1.0 mm × 1.0 mm).

For preprocessing and statistical analyses, the acquired images were converted from the Siemens DICOM format to the NIFTI format using the dcm2nii utility (version 2013) (Li, Morgan, Ashburner, Smith, & Rorden, 2016). Functional images (EPIs) were spatially realigned to the mean EPI image (using second degree B‐spline interpolation) followed by slice‐timing correction (relative to the middle slice). The mean EPI was then co‐registered to the structural image. Using SPM12's unified segmentation/normalization algorithm, the structural image was segmented to distinguish white from gray matter and then warped to match a standard Montreal Neurological Institute (MNI) template brain image. The deformation fields estimated from this segmentation/normalization procedure were applied to all EPIs to transform them into standard MNI space (normalization) followed by resampling to a voxel size of 2 mm × 2 mm × 2 mm (fourth degree B‐spline interpolation). The normalized EPIs were smoothed with an isotropic 8 mm full‐width‐at‐half‐maximum (FWHM) Gaussian kernel. For statistical analyses involving the explicit estimation of the timing of the hemodynamic response (see below), a concern was that specific preprocessing steps, such as the slice‐timing correction and smoothing, could distort inter‐voxel timing relationships (e.g., Kamitani & Sawahata, 2010). Therefore, for these time‐sensitive analyses, we created a duplicate dataset that was preprocessed as described above but omitted the slice‐time correction step and used a smaller smoothing kernel of 6 mm (rather than 8 mm). No unwarping (e.g., Andersson, Hutton, Ashburner, Turner, & Friston, 2001) was performed on either dataset during preprocessing to minimize potential distortions of timing information.

Following preprocessing, datasets from three individuals were excluded from further analysis: one due to technical errors in recording responses; another due to an incomplete scan due to technical delays; and one for low overall accuracy (~50%). For all remaining participants, fewer than 5% of the ~2,600 images acquired (4 runs × ~650 images/run) were affected by large head movements defined here as a frame‐wise displacement greater than 0.5 mm between consecutive images (Power, Barnes, Snyder, Schlaggar, & Petersen, 2012) (assessed using the ArtRepair software [Mazaika, Hoeft, Glover, & Reiss, 2009]).

### 2.7 fMRI statistical analysis

All statistical analyses were conducted within a mass‐univariate framework where the evoked hemodynamic response at each voxel was independently estimated using a general linear model (GLM). Two different basis functions were used for the analysis. As with prior studies, the BOLD effects of the coherence modulation were modeled using the canonical Hemodynamic Response Function (HRF) basis (Friston et al., 1998; Josephs, Turner, & Friston, 1997; Worsley & Friston, 1995) (on the slice‐time corrected EPIs). However, to explicitly account for the role of timing, the BOLD effects of the MvT modulation were modeled using the Finite Impulse Response (FIR) basis function (Boynton, Engel, Glover, & Heeger, 1996; Glover, 1999; Goutte, Nielsen, & Hansen, 2000; Ollinger, Corbetta, & Shulman, 2001; Ollinger, Shulman, & Corbetta, 2001) (on EPIs without slice‐time correction). For both basis functions, the trial onset was defined at the stimulus onset.

- *Coherence modulation (canonical HRF basis)*: To assess the effect of coherence modulation on perceptual processes that are invariant to action selection/execution, correct trials with the left and right index‐finger responses were each modeled using a single regressor (collapsing across the Mov factors) with a parametric regressor to assess modulation by coherence. For simplicity, parametric modulation was assumed to be linear, and the coherence levels {Coh_Low_, Coh_Med_, Coh_High_} were coded as [+1, 0, −1], respectively (Philiastides & Sajda, 2007). Since the coherence‐calibrated RTs had a small range (300 ms), trial duration was set to 0 ms.
- *Movement time modulation (FIR basis)*: To identify MvT‐related effects, each of the 12 conditions (i.e., Hand {Left, Right} × Coherence {Coh_Low_, Coh_Med_, Coh_High_} × Mov {Mov_Short_, Mov_Long_}) was represented by separate regressors in the GLM. For each condition, a 24 s period following stimulus onset was modeled with one regressor (“stick” function) at each of 16 discrete time points at intervals of 1.5 s. The value of 1.5 s was heuristically selected to strike a balance between the number of regressors required per condition while being shorter than the TR (2.2 s).

Independent of the basis function used, all estimated first‐level models shared the following properties. The regressors of interest only modeled correct trials. Additional regressors of non‐interest were included to account for incorrect trials and the feedback period. The six head‐movement parameters estimated during spatial realignment (i.e., translation and rotation relative to the *X*, *Y*, *Z* axes) were included as covariates to account for head‐movement effects. To increase statistical power, images from all runs were concatenated and analyzed as a continuous time‐series in the GLM with additional regressors to account for inter‐run differences. The time‐series at each voxel were high‐pass filtered (1/128 Hz) to remove slow trends. First‐level model estimation was restricted to voxels contained within a full‐brain mask estimated during normalization.

For second‐level statistical analyses, analyses were restricted to voxels contained within a functionally defined mask of voxels that exhibit task‐related activity changes. This task‐mask consisted of all voxels where the mean activity on the left and right index finger response trials (estimated using the canonical HRF basis function) was significantly greater than zero (second‐level *t* test, *p* < .05 FWE, corrected at the voxel level). The boundaries of the task‐mask are displayed in the visualization of the second‐level analyses in Section 3. Unless noted otherwise, voxel‐level activity across the Left/Right conditions was averaged together based on each voxel's lateralized location relative to the moving finger, that is, contralateral or ipsilateral. For all statistical maps, contralateral activity was displayed over the left hemisphere.

Contrasts were performed at the individual level (i.e., first‐level), and these contrast images (without any additional smoothing) were used for group (i.e., second‐level) statistics. Second‐level statistics were only assessed at voxels contained within the task mask. Due to our interest in obtaining spatial maps, all second‐level statistics reported here were corrected for multiple comparisons at the threshold of *p* < .05 FWE cluster‐corrected with a cluster‐forming threshold determined at *p* < .0001 (uncorrected). For exploratory purposes, the conjunction between activity maps was assessed based on the minimum‐T criterion (Nichols, Brett, Andersson, Wager, & Poline, 2005). For clarity, statistical tables only report clusters and peak‐coordinates from the contralateral hemisphere.

Statistical maps were rendered on the Conte69 cortical surface (Van Essen, Glasser, Dierker, Harwell, & Coalson, 2012) using the Workbench software (wb_command 1.2.2, 2016‐07‐18; Marcus et al., 2011).

### 2.8 FIR time‐series analysis

The first‐level model estimation with the FIR basis produced 192 beta images for the 12 conditions (12 conditions × 16 time points). For computational convenience, the beta‐values for each voxel, time‐point, and condition were extracted into a numerical matrix of size *N* × *T* per condition, where *N* = number of voxels within the task‐mask (~90,000 voxels), and *T* = number of modeled time‐points.

Even though 16 time points (24 s) were modeled, we restricted our analysis to a shorter period of *T* = 9 time points (12 s) to exclude low amplitude hemodynamic fluctuations at the end of the modeled period. The shorter 12 s period was selected based on the following theoretical approximation: The estimated duration of a full trial is approximately 3 s. Convolving a boxcar function of 3 s duration with the canonical HRF produces a predicted hemodynamic response with an amplitude that peaks at ~6.6 s following stimulus onset. By treating 6 s as the approximate mid‐point of task‐relevant hemodynamic changes on a given trial, we restricted the subsequent analysis to a 2 × 6 s = 12 s period following stimulus‐onset.

These voxel × time beta‐value matrices from each condition were used to analyze the (a) hemodynamic states at each time‐point (i.e., columns of the matrix), and (b) voxel‐specific dynamics (i.e., rows of the matrix).

### 2.9 Hemodynamic state and Euclidean distance

**FIGURE 2:**
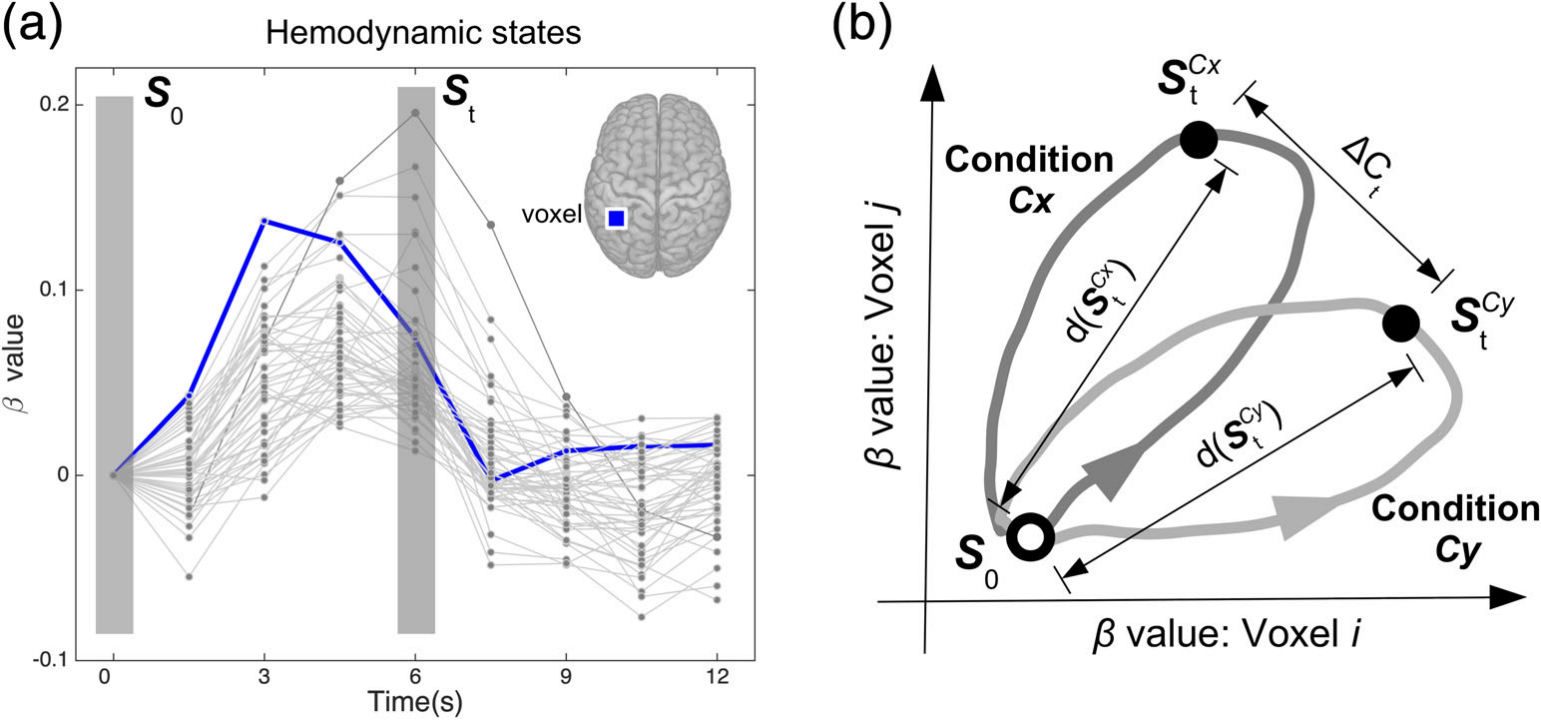
Hemodynamic states. (a) The beta‐values at all analyzed voxels at time *t* define the hemodynamic state ***S***_*t*_ (shaded box). For illustration, beta values at a subset of voxels are shown where each line corresponds to the beta‐values at one voxel. The thick line is an example of beta‐values at the voxel marked in the inset. (b) Geometric representation of the hemodynamic state ***S***_*t*_ (solid black dot) as a point in an *n*‐dimensional space where each voxel defines one dimension, here shown for two voxels. The sequence of coordinates (i.e., states) occupied over time for each condition is shown as a directed curve (dark gray for condition *Cx*, light gray for condition *Cy*)

Each column of the voxel × time matrices described above were used to obtain a snapshot of the instantaneous activity across the brain (i.e., the hemodynamic state) at each time‐point. Specifically, the instantaneous hemodynamic state at time *t* (denoted as ***S***_***t***_) for each condition was defined as a vector of beta‐values ***S***_***t***_ ***=*** [*β*_1,*t*_ *β*_2,*t*_…*β*_*N*,*t*_], where *β*_*v*,*t*_ denotes the beta‐value at voxel *v* at time *t* (Figure 2a). Each of the 12 conditions was associated with nine states corresponding to each of the *T* time points. Therefore, intercondition differences should manifest as a difference between the corresponding hemodynamic states at one or more time‐points independent of how the task‐specific networks are organized.

To quantify inter‐state differences, we formulated the hemodynamic states and their relationships in geometric terms (Figure 2b). The hemodynamic state is treated as a point in a multidimensional space where each voxel defines one dimension, similar to the typical formulation used in Multivariate/voxel Pattern Analysis (MVPA) (Cohen et al., 2017; Haxby, Connolly, & Guntupalli, 2014; Norman, Polyn, Detre, & Haxby, 2006). The Euclidean distance between two states was used as a measure of the difference in activity (as indexed by the beta‐values) between those states.

The overall activity level of a particular state ***S***_*t*_ in condition *C* was defined as its Euclidean distance to the starting state ***S***_0_ of that same condition, namely,

$$d\left(S_{t}\right)=\sqrt{\sum_{v=1}^{N}{\left(\beta_{v,t}–\beta_{v,0}\right)}^{2}}$$

The relative difference in activity (i.e., Euclidean distance) between a hemodynamic state in condition *Cx* ($S_{t}^{\mathrm{Cx}}$) to the corresponding state in condition *Cy* at the same time *t* ($S_{t}^{\mathrm{Cy}}$) was computed as

$$\Delta C_{t}=\sqrt{\sum_{v=1}^{N}{\left(\beta_{v,t}^{\mathrm{Cx}}–\beta_{v,t}^{\mathrm{Cy}}\right)}^{2}}$$

As illustrated in Figure 2b, the two states ***S***_*t*_^*Cx*^ and ***S***_*t*_^*Cy*^ are different from each other in relative activity (i.e., Δ*C*_*t*_ > 0) as voxel *j* has a relatively higher activity than voxel *i* for condition *Cx* while the opposite is true for *Cy*. Nevertheless, both states can have the same Euclidean distance to the start state ***S***_0_ (i.e., *d*(***S***_*t*_^*Cx*^) = *d*(***S***_*t*_^*Cy*^)).

The Euclidean distance between any two states, whether within or between conditions, was always a single scalar value. All measures were averaged across responding hand.

### 2.10 Peak amplitude and peak time

The hemodynamic changes at each voxel were summarized using two parameters related to the activity peak, namely, the peak amplitude and the peak time as described below.

The voxel × time beta‐value matrix for each condition (described above) provided access to the beta time‐series at each voxel *v* for each condition *C*, that is, [*β*_1,*t*_ *β*_2,*t*_…*β*_*N*,*t*_]. We qualitatively verified that the beta time‐series at most voxels showed a single‐peaked shape across conditions and participants (as in Figure 2a). This time‐series was summarized with two parameters: the peak amplitude (i.e., the maximum beta value of the time series), and the peak time (i.e., the earliest time‐point at which the peak amplitude is reached).

For each condition, the peak amplitude (denoted as ${\bar{\beta }}_{v}$) and peak time at each voxel (denoted as ${\bar{t}}_{v}$) were written to the corresponding voxel location of an empty image having the same dimensions as the beta images and then smoothed (6 mm FWHM). These summary images were used to calculate intercondition contrasts in peak amplitudes and peak times per individual, and these contrast images were statistically evaluated at the group level.

### 2.11 Normalized difference in peak amplitude and peak time

To assess the relative magnitude of intercondition differences in peak amplitude/time across different voxels, these differences were normalized similar to the coefficient of variation (i.e., [*SD*]/mean). The normalized difference of the peak amplitude and peak time differences between conditions *Cx* and *Cy* at voxel *v* (i.e.,${\bar{\beta }}_{v}^{\mathrm{Cx}}-{\bar{\beta }}_{v}^{\mathrm{Cy}}$ and ${\bar{t}}_{v}^{\mathrm{Cx}}-{\bar{t}}_{v}^{\mathrm{Cy}}$) was defined as follows and expressed as a percentage

$$\rho_{\beta }=100\times \frac{{\bar{\beta }}_{v}^{\mathrm{Cx}}-{\bar{\beta }}_{v}^{\mathrm{Cy}}\;}{{\bar{\beta }}_{v}^{\mathrm{Cx}}+{\bar{\beta }}_{v}^{\mathrm{Cy}}\;}\;\text{and}\;\rho_{t}=100\times \frac{{\bar{t}}_{v}^{\mathrm{Cx}}-{\bar{t}}_{v}^{\mathrm{Cy}}}{{\bar{t}}_{v}^{\mathrm{Cx}}+{\bar{t}}_{v}^{\mathrm{Cy}}}$$

These values were computed at the first level using the peak amplitude and peak time summary images described above.

### 2.12 M1 region of interest

An M1 region of interest was defined using a combination of functional and anatomical criteria. A variant of the coherence‐modulation model was used to define M1 functionally. The correct trials produced with the left and right index fingers were modeled as separate regressors with a corresponding parametric regressor to account for movement‐time effects, namely, Mov_Short_ and Mov_Long_ (collapsed across all coherence levels). To functionally identify M1 for each hemisphere, the set of all voxels where the parametric modulation showed a significant parametric increase for the contralateral index finger responses (second‐level *t* test, *p* < .05 FWE, corrected at the voxel level) was identified. These voxels were then masked with the Human Motor Area Template (HMAT) atlas (Mayka, Corcos, Leurgans, & Vaillancourt, 2006). The coordinate of the local maximum within this masked area that was closest to (a) the “hand‐knob formation” at the precentral gyrus (Yousry, 1997) and (b) functionally identified coordinates for finger tapping (based on the meta‐analysis by Witt, Laird, & Meyerand, 2008) was defined as the reference coordinate for M1 in each hemisphere (MNI coordinates for right M1: [34–22 60], left M1: [−38–22 62]). A sphere of 4 mm radius (33 voxels) centered at these coordinates was defined as the M1 region of interest.

## 3 RESULTS

### 3.1 Behavioral validation

Our experiment was designed to modulate (a) response times by varying the motion coherence of the RDK stimuli, and (b) movement times by varying the number of button‐presses (Figure 1b).

Consistent with this first goal, increasing coherence was accompanied with a systematic decrease in the mean RT (Coh_Low_: 899 ± 8 ms; Coh_Med_: 836 ± 5 ms; Coh_High_: 750 ± 11 ms) (mean ± within‐subject *SEM*) [ANOVA, *F*_(2,58)_ = 75.19, *p* < .0001]. There was also a systematic increase in the mean accuracy of the decisions with increasing coherence (Coh_Low_: 79.35 ± 0.97%; Coh_Med_: 87.91 ± 0.56%; Coh_High_: 93.76 ± 1.02%) [ANOVA, *F*_(2,58)_ = 68.74, *p* < .0001].

Consistent with the goal of modulating movement time (MvT), the mean time (averaged across hands) to execute 3 button‐presses(Mov_Short_) (542 ± 13 ms) was smaller than to execute 8 button‐presses (Mov_Long_)(1,496 ± 13 ms) [paired *t*_(29)_ = 36.38, *p* < .0001].

These behavioral differences were a pre‐condition to evaluate the readout of the corresponding neural dynamics in the BOLD signal.

### 3.2 Response time and movement time have dissociable hemodynamic effects

To read out response‐locked neural activity in the BOLD activity, the independent modulation of RT and MvT at the neural level should have dissociable effects on the hemodynamic responses. In our paradigm, the RDK coherence modulated the RT (i.e., the time from the stimulus to the first button press) *before* the MoveOff stimulus could influence the MvT (i.e., the time between the first and last button‐press). These sequential effects predicted an asymmetry relative to the stimulus onset. The timing of the neural activity differences between the conditions <Coh_Low_, Mov_*x*_> and <Coh_High_, Mov_*x*_> should be unaffected by whether Mov_*x*_ was equal to Mov_Short_ or Mov_Long_. However, the neural differences between the conditions <Coh_*x*_, Mov_Long_> and <Coh_*x*_, Mov_Short_> should occur later when Coh_*x*_ was equal to Coh_Low_ (i.e., high RT) rather than Coh_High_ (i.e., low RT). We tested whether this predicted asymmetry was detectable in the hemodynamic states constructed from the beta‐value time‐series estimated with the FIR basis function (see Figure 2, Section 2).

**FIGURE 3:**
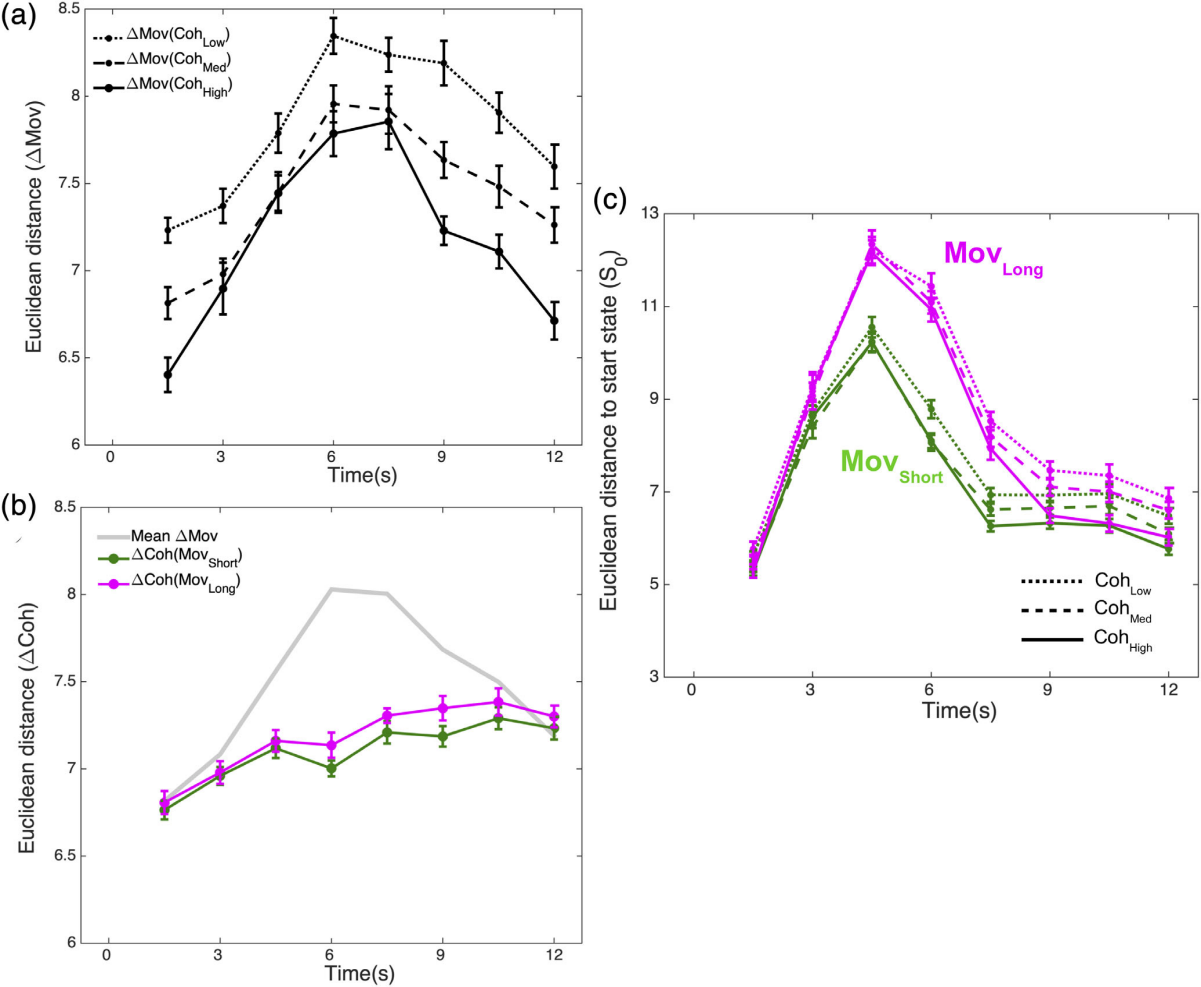
Intercondition state differences. (a) Mean Euclidean distance between <Coh_*x*_, Mov_Long_> and <Coh_*x*_, Mov_Short_> for each value of Coh_*x*_ indicated as ΔMov(Coh_*x*_) (error bars are within‐subject standard error of the mean [Morey, 2008]). (b) Mean pair‐wise Euclidean distance between <Coh_*x*_, Mov_A_> and <Coh_*y*_, Mov_A_> for different values of Coh_*x*_ and Coh_*y*_ when Mov_A_ = Mov_Short_ (green line) and when Mov_A_ = Mov_Long_ (magenta line). The gray line indicates the mean value of ΔMov (from panel (a)) averaged across all coherence values. (c) Mean Euclidean distance to the start state ***S***_0_ for all <Coh, Mov> combinations

Figure 3a shows the Euclidean distance between the time‐resolved states for <Coh_*x*_, Mov_Short_> and <Coh_*x*_, Mov_Long_> (denoted as ΔMov) for each of the three possible values of Coh_*x*_ (i.e., Coh_Low_, Coh_Med_, Coh_High_). Coherence robustly modulated the magnitude of ΔMov with a weaker modulation of ΔMov timing (ANOVA, Coh {Coh_Low_, Coh_Med_, Coh_High_} × time {8 time bins}; Coh*Time: *F*_(14,406)_ = 1.73, *p* = .048; Coh: *F*_(2,58)_ = 10.46, *p* = .0001; Time: *F*_(7,203)_ = 32.52, *p* < .0001).

The corresponding modulatory relationship was absent for the mean pair‐wise Euclidean distance between the states for Coh_Low_, Coh_Med_, and Coh_High_ (denoted as ΔCoh) for each Mov level (Figure 3b). ΔCoh for Mov_Short_ and for Mov_Long_ steadily increased over the entire modeled period but was not modulated by the Mov level (ANOVA, Mov {Mov_Short_, Mov_Long_} × Time {8 time bins}; Mov*Time: *F*_(7,203)_ = 0.39, *p* = .91; Mov: *F*_(1,29)_ = 2.26, *p* = .14; Time: *F*_(7,203)_ = 15.39, *p* < .0001).

Furthermore, the manner in which ΔMov changed over time (i.e., with a single peak when averaged across Coh levels) was robustly different from how ΔCoh changed over time (i.e., steadily increasing when averaged across Mov levels) (ANOVA, Modulation {ΔCoh, ΔMov} × Time {8 time bins}; Modulation*Time: *F*_(7,203)_ = 33.285, *p* < .0001; Modulation: *F*_(1,29)_ = 54.05, *p* < .00001; Time: *F*_(7,203)_ = 25.85, *p* < .00001). Taken together, these findings (i.e., Coh modulated ΔMov but Mov did not modulate ΔCoh) confirmed the asymmetric relationship between Coh and Mov predicted by their sequential effects.

To clarify whether the observed asymmetry of the Coh/Mov relationships was indeed linked to differences in the timing of neural activations, we assessed a measure of overall activity that ignored inter‐voxel timing differences, namely, the Euclidean distance of each state to the start state ***S***_0_. With this measure, a simple prediction was that the overall activity for <Coh_Low_, Mov_*x*_> (i.e., high RT) would be shifted later in time as compared to <Coh_High_, Mov_*x*_> (i.e., low RT), irrespective of whether Mov_*x*_ was Mov_Short_ or Mov_Long_. For all Coh × Mov combinations (Figure 3c), there was a sharp initial increase in overall activity (relative to ***S***_0_) followed by a subsequent decrease. For all coherence levels, Mov_Long_ was associated with a greater overall activity than Mov_Short_. However, this influence of Mov was not modulated by coherence contrary to the above prediction. Specifically, the statistical interaction of Mov and Coh was not significant in a 3‐way ANOVA with factors Coh {Coh_Low_, Coh_Med_, Coh_High_} × Mov {Mov_Short_, Mov_Long_} × Time {8 time bins (excluding t_o_)} (Coh*Mov*Time: *F*_(14,406)_ = 0.67, *p* = .80; Coh*Mov: *F*_(2,58)_ = 0.84, *p* = .44; Coh*Time: *F*_(14,406)_ = 3.98, *p* < .0001; Mov*Time: *F*_(7,203)_ = 59.34, *p* < .0001; Mov: *F*_(1,29)_ = 175.41, *p* < .0001; Coh: *F*_(2,58)_ = 23.38, *p* < .0001; Time: *F*_(7,203)_ = 137.86, *p* < .0001).

In summary, the time‐resolution of the BOLD signal was sufficient to detect neural differences at the time scales of our paradigm even without distinguishing between the functional networks modulated by stimulus coherence (Coh) and by the number of button‐presses(Mov). However, the timing differences linked to the Coh/Mov relationships were only evident in multivariate inter‐voxel comparisons and not by measures of overall activity relative to ***S***_0_ (see Figure 2). We next turned to map the spatial locations of these response‐locked differences.

### 3.3 Mapping response‐locked activity reveals functional distinctions

**FIGURE 4:**
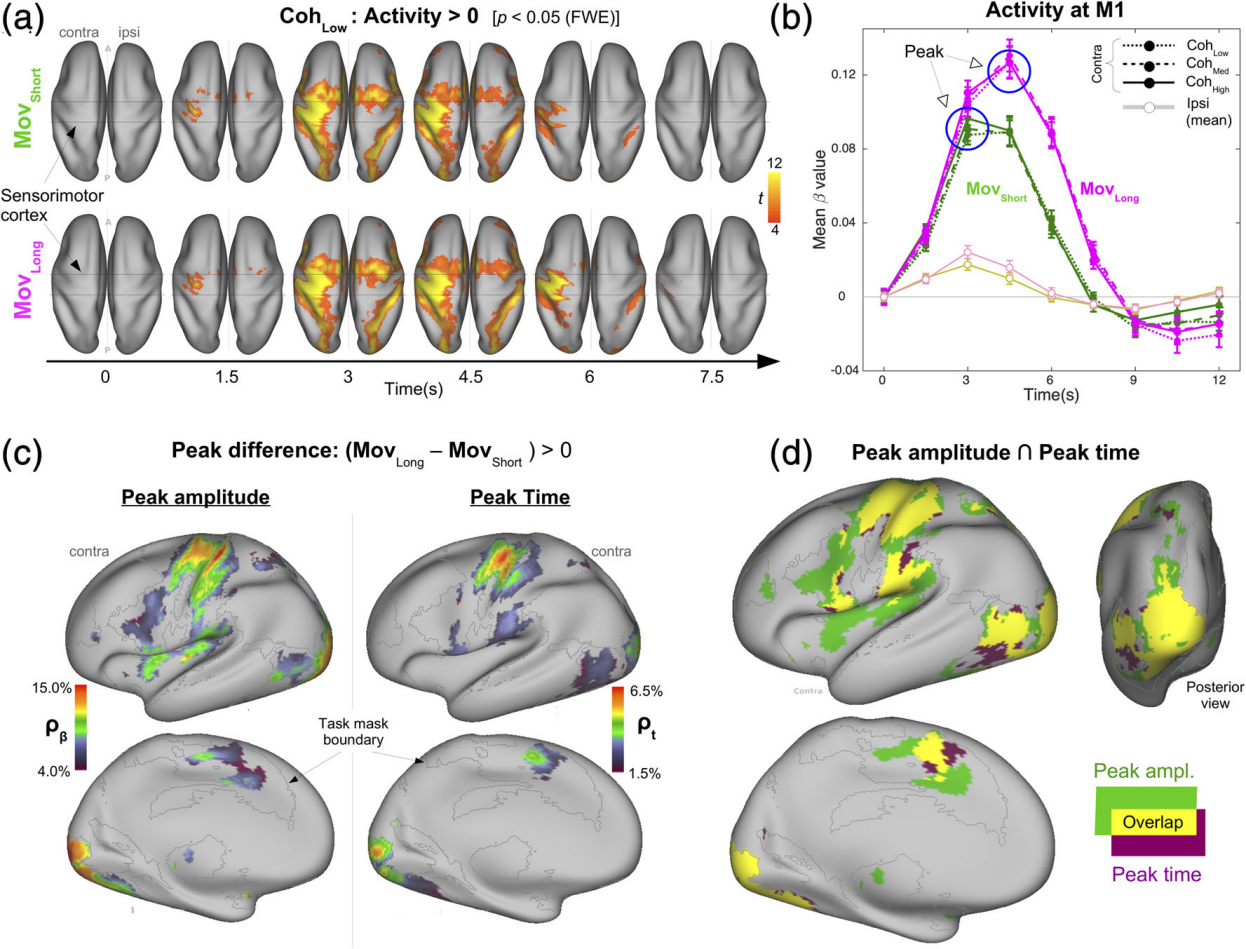
Mapping movement time. (a) Distribution of voxels showing statistically significant activity increases following movement onset for <Coh_Low_, Mov_Short_> (upper panel) and <Coh_Low_, Mov_Long_ > (lower panel) (*p* < .05, voxel‐wise FWE). The area between horizontal lines indicates the approximate location of the motor/somatosensory cortex. (b) Activity over time for different <Coh, Mov> combinations at contralateral M1 (dark lines with filled dots, magenta and green), and ipsilateral M1 averaged over coherence values (light lines/open dots). Across coherence values, the peak amplitude for Mov_Long_ was higher than for Mov_Short_ and was also reached at a later time. (c) Map of voxels over contralateral hemisphere where peak activity for Mov_Long_ was significantly greater than for Mov_Short_ in peak amplitude (left column) and in peak time (right column) (*p* < .05, FWE‐cluster corrected, cluster‐forming threshold for peak amplitude = 52 voxels, for peak time = 41 voxels). Colors indicate normalized differences in peak amplitude/time at these voxels. (d) Relationship between maps of movement‐related peak amplitude (green) and peak time (magenta) differences. The maps show considerable overlap (yellow) [minimum cluster size = 41 voxels] but also considerable nonoverlapping regions

The readout of the response‐locked neural differences between <Coh_*x*_, Mov_Short_> and <Coh_*x*_, Mov_Long_> in the stimulus‐locked BOLD signal could take different forms across the brain. For example, the mean BOLD activity for <Coh_Low_, Mov_Long_> was significantly greater than zero over a longer period (until 7.5 s) than for <Coh_Low_, Mov_Short_> (until 6 s) at several voxels (Figure 4a). Nevertheless, the activity profiles at these voxels could differ from each other, depending on their *functional* origin (Ploran et al., 2007). In our paradigm, a basic functional difference between <Coh_*x*_, Mov_Short_> and <Coh_*x*_, Mov_Long_> was related to movement production (i.e., 3 versus 8 button‐presses). Therefore, we used the activity profile at the primary motor cortex (M1) as a template to map the potential functional origin of the response‐locked differences.

Across <Coh, Mov> conditions, the activity at M1 contralateral to the responding hand (Figure 4b) was robustly modulated by Mov (i.e., Mov*Time) and coherence did not modulate this relationship (Mov*Coh*Time) (3 way‐ANOVA, Mov {Mov_Short_, Mov_Long_} × Coh {Coh_Low_, Coh_Med_, Coh_High_} × Time {9 time‐bins}; Mov*Coh*Time: *F*_(16,464)_ = 0.65, *p* = .84; Mov*Time: *F*_(8,232)_ = 54.10, *p* < .0001; Coh*Time: *F*_(16,464)_ = 3.79, *p* < .0001; Mov*Coh: *F*_(2,58)_ = 0.86, *p* = .43; Mov: *F*_(1,29)_ = 55.88, *p* < .0001; Coh: *F*_(2,58)_ = 2.08, *p* = .13; Time: *F*_(8,232)_ = 86.33, *p* < .0001). Consistent with the additive effect of repetitive button presses, the peak beta‐value for Mov_Long_ (8 button presses) was larger and occurred later (relative to stimulus onset) than for Mov_Short_ (3 button presses). This peak‐based pattern of differences was used as a functional template.

Activity at a voxel was categorized as being “like M1” (i.e., potentially having a movement‐related origin) if the *peak amplitude* and *peak time* for <Coh_*x*_, Mov_Long_> were significantly greater than for <Coh_x_, Mov_Short_> (averaged across Coh levels). Voxels that satisfied only the peak amplitude criterion or only the peak time criterion were categorized as being “unlike M1” (i.e., having a non‐movement origin). Due to the low activity at ipsilateral M1 (Figure 4b), which was expected given the relative simplicity of the required unilateral responses, we restricted our subsequent analyses to the contralateral hemisphere.

The peak amplitude criterion and the peak time criterion were each satisfied over an extensive set of regions (Figure 4c, Tables S1 and S2 **)**. More voxels satisfied the peak amplitude criterion (Figure 4c, left panel) than the peak time criterion (Figure 4c, right panel). Furthermore, the maximum normalized difference in peak amplitude (~15%) was more than twice the corresponding maximum for peak time (~6%). Large normalized differences in peak amplitude and in peak time were concentrated over M1, S1, and the occipital cortex.

The (binary) maps specific to the peak amplitude and the peak time criteria were overlaid to identify voxels based on the like/unlike‐M1 categorization rule (Figure 4d, Table 1). The overlap of the peak amplitude and peak time maps (yellow areas) included M1, S1, and S2 with additional overlaps at the occipital cortex. Notably, several regions were unlike M1. Only the peak amplitude criterion (green areas) was satisfied at caudal SMA, anterior ventral premotor cortex (prePMV) in the vicinity of area 44/45, posterior insula, and cingulate gyrus. Despite the poor time resolution of the hemodynamic signal, regions that met the peak time criterion (magenta areas) were not strictly a subset of regions satisfying the peak amplitude criterion. There were large clusters over preSMA where only the peak time criterion was satisfied.

**TABLE 1:** Overlap peak amplitude and peak time maps (contralateral hemisphere)

Cluster size	Peaks					
	*x*	*y*	*z*	*T* value	Anatomical region	Location (BA)
8,069	−24	−90	−6	16.58	Inferior occipital gyrus	hOc3v [V3v] (19)
	−12	−98	2	13.14	Middle occipital gyrus	hOc1 [V1] (17)
	−32	−72	−10	11.33	Fusiform gyrus	FG1/hOc4v [V4(v)] (37)
	−34	−86	8	8.98	Middle occipital gyrus	hOc4lp/hOc4la (19)
	−44	−64	0	8.81	Middle temporal gyrus	hOc5 [V5/MT] (37)
	−24	−96	20	7.67	Superior occipital gyrus	hOc3d [V3d]/hOc4d [V3A] (19)
	−36	−56	−20	7.32	Fusiform gyrus	FG2 (37)
	−28	−78	22	5.58	Middle occipital gyrus	(19)
3,164	−40	−18	50	16.04	Precentral gyrus	**M1 (**4a/4p, 3)
	−34	−24	68	11.78	Precentral gyrus	**M1** (4)
	−50	−16	18	9.83	Postcentral gyrus	OP3 [VS]/OP4 [PV] (41)
	−42	−34	48	7.13	Postcentral gyrus	(2/3b)
	−60	−34	22	5.75	Superior temporal gyrus	PFcm (IPL)/PF/IPL (40)
	−34	−24	18	4.88	Insula lobe	OP2 [PIVC]/Ig1 (OP2)
	−58	−26	40	4.55	Supramarginal gyrus	PFt (IPL)/2 (40)
509	−6	−2	56	11.5	Dorsal medial frontal cortex	**SMA/preSMA** (6)
366	−12	−16	8	9.71	Thalamus	Thal: Prefrontal/Thal: Temporal
237	−56	2	2	8.15	Frontal operculum	TE (3)/TE (1.2)
	−58	10	28	6.85	Precentral gyrus	**vPMC (**44/45, 6)

*Note*: Clusters identified at a threshold of *p* < .05 (FWE, cluster‐corrected), cluster threshold = 41 voxels (*p* < .0001 uncorr.). Peak locations are in MNI coordinates (min distance between intra‐cluster peaks = 16 mm). Numbers in parentheses in the last column indicate the Brodmann area (BA).

In summary, irrespective of functional origin, the activity differences revealed by the map in Figure 4d meet the definition of being response‐locked, namely, they followed *after* the first button‐press. Applying a “like/unlike‐M1” functional template to these response‐locked differences revealed that several regions had an activity profile like M1 (as expected), but several regions had an activity profile that was unlike M1 in different ways. To clarify these functional distinctions revealed by response‐locking, we next compared it to a conventional stimulus‐locked view of these data.

### 3.4 Stimulus‐locked activity: Coherence‐modulated networks

**FIGURE 5:**
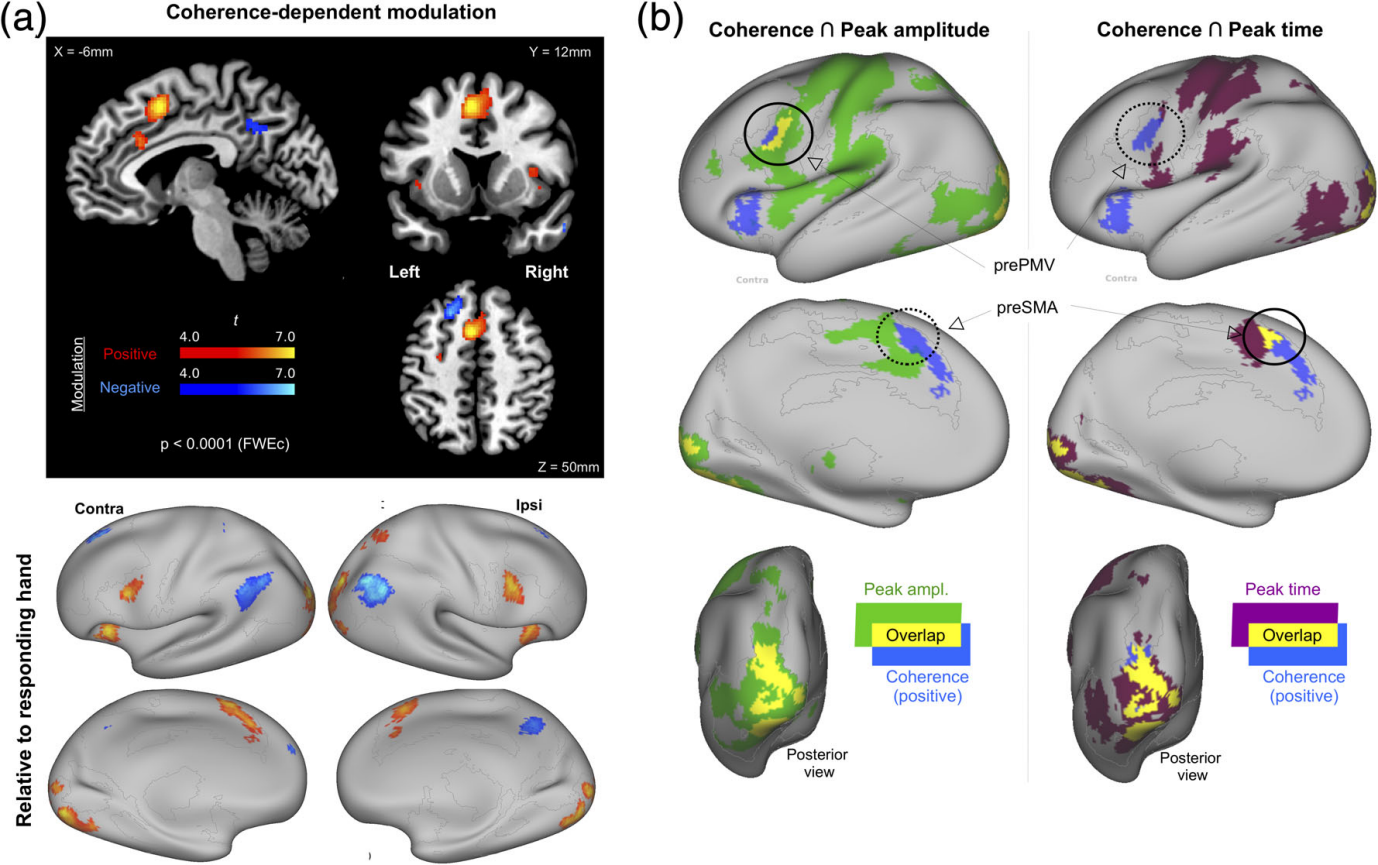
Stimulus‐modulation. (a) Map of voxels showing significant modulation with a change in stimulus coherence (*p* < .05, FWE‐cluster corrected, cluster‐forming threshold identified at *p* < .0001, uncorrected). Regions shown in red show significant positive modulation, namely, increases with increasing RT (or decreasing coherence [see inset]) while regions in blue show significant negative modulation, namely, reductions in activity with decreasing RT (i.e., increasing coherence). (b) Relationship between coherence‐modulated regions (positive modulation) and (i) peak amplitude map (left column) and (ii) peak time (right column). Overlaps (yellow) have a minimum cluster size of 41 voxels

Coherence‐modulated activity relative to the stimulus onset was estimated using the canonical HRF basis function. When averaged across Mov levels and responding hand, changes in stimulus coherence modulated activity negatively in some regions and positively in others (Figure 5a upper, Tables 2 and 3). Since stimulus identification precedes action selection, the modulation is displayed without flipping the data relative to the responding hand. Parametric decreases in coherence (i.e., increases in RT) were associated with a reduction in mean activity (i.e., negative modulation) at the left dorsolateral prefrontal cortex (dLPFC), bilateral angular/supramarginal gyrus, precuneus, and the thalamus. However, coherence decreases were associated with increased activity (i.e., positive modulation) at multiple occipital and posterior parietal regions, at the cingulo‐opercular regions (middle cingulate cortex, anterior insula bilaterally), and bilateral precentral/inferior frontal gyrus rostral to the ventral premotor cortex (prePMV), SMA, and pre‐SMA.

**TABLE 2:** Coherence positive modulation (contralateral hemisphere)

Cluster size	Peaks					
	*x*	*y*	*z*	*T* value	Anatomical region	Location (BA)
276	−46	2	32	7.86	Precentral gyrus	**prePMV** (44, 6)
1,309	−26	−84	14	7.69	Middle occipital gyrus	hOc4lp (19)
	−26	−88	−10	6.92	Inferior occipital gyrus	hOc4v [V4(v)]/hOc3v [V3v] (19)
	−14	−100	−2	5.63	Calcarine gyrus	hOc1 [V1]/hOc2 [V2] (17)
	−22	−66	38	5.26	Superior occipital gyrus	hIP3 (IPS) (7)
	−28	−74	0	5.22	Fusiform gyrus	FG1 (19)
802	−6	10	50	7.43	Dorsal medial frontal cortex	**preSMA** (6)
	−8	24	30	6.16	Anterior cingulate cortex	aMCCd (24)
336	−28	26	0	7.18	Insula lobe	
	−42	16	6	5.51	Insula lobe	(44)
126	−30	−2	72	5.78	Precentral gyrus	**dPMC** (6)

*Note*: Clusters were identified at a threshold of *p* < .05 (FWE, cluster‐corrected), cluster threshold = 92 voxels (*p* < .0001 uncorr.). Peak locations are in MNI coordinates (min distance between intracluster peaks = 16 mm). Numbers in parentheses in the last column indicate the Brodmann area (BA).

**TABLE 3:** Coherence negative modulation (contralateral hemisphere)

Cluster size	Peaks					
	*x*	*y*	*z*	*T* value	Anatomical region	Location (BA)
242	−20	26	50	6.34	Middle frontal gyrus	**DPLFC**(8)
50	−22	4	−10	6.18	Putamen	
327	−54	−62	36	6.02	Angular gyrus	PGa (IPL)/PFm (IPL) (40)
245	−14	−48	34	5.87	MCC/precuneus	(31)
56	−16	56	12	5.11	Superior frontal gyrus	Fp1 (10)
242	−20	26	50	6.34	Middle frontal gyrus	**DPLFC** (8)
50	−22	4	−10	6.18	Putamen	
327	−54	−62	36	6.02	Angular gyrus	PGa (IPL)/PFm (IPL) (40)
245	−14	−48	34	5.87	MCC/precuneus	(31)
56	−16	56	12	5.11	Superior frontal gyrus	Fp1 (10)

*Note*: Clusters were identified at a threshold of *p* < .05 (FWE, cluster‐corrected), cluster threshold = 50 voxels (*p* < .0001 uncorr.). Peak locations are in MNI coordinates (min distance between intracluster peaks = 16 mm). Numbers in parentheses in the last column indicate the Brodmann area (BA).

Even though the stimulus‐locked and response‐locked activity maps were estimated with different basis functions, for exploratory purposes, we compared them to assess if the hypothesized increase in sensitivity with response‐locking revealed any additional information about the stimulus‐locked effects. For this comparison, the functional activity estimates for the left−/right‐hand responses were converted to a contralateral/ipsilateral reference (Figure 5a, lower). Furthermore, we focused only on regions showing increased activity with increasing RT (i.e., positive modulation) to allow comparison with regions categorized based on response‐locked increases in peak‐amplitude/time with increasing MvT. Binary maps of these positively‐modulated regions were overlaid with the peak amplitude and peak time maps, as shown in Figure 5b (Table 4).

**TABLE 4:** Overlap (contralateral hemisphere)

Cluster size	Peaks					
	*x*	*y*	*z*	*T* value	Anatomical region	Location (BA)
*Amplitude and coherence*						
1,201	−24	−88	−6	8.56	Inferior occipital gyrus	hOc3v [V3v]/hOc4v [V4(v)] (19)
	−28	−84	16	7.22	Middle occipital gyrus	hOc4lp (19)
	−28	−70	−10	7.15	Fusiform gyrus	FG1/hOc4v [V4(v)] (37)
	−12	−100	0	6.4	Middle occipital gyrus	hOc1 [V1]/hOc2 [V2] (17)
83	−48	2	32	5.57	Precentral gyrus	**prePMV** (44, 6)
						
*Time and coherence*						
1,115	−24	−88	−6	8.56	Inferior occipital gyrus	hOc3v [V3v]/hOc4v [V4(v)] (19)
	−16	−100	4	6.39	Middle occipital gyrus	hOc3d [V3d]/hOc2 [V2] (18)
	−30	−72	−8	5.86	Fusiform gyrus	FG1/hOc4v [V4(v)] (19)
	−26	−96	20	5.02	Middle occipital gyrus	hOc4d [V3A]/hOc3d [V3d] (19)
149	−8	12	56	5.29	Dorsal medial frontal cortex	**SMA/preSMA** (6)

*Note*: Clusters were identified at a threshold of *p* < .05 (FWE, cluster‐corrected), cluster threshold = 41 voxels (*p* < .0001 uncorr.). Peak locations are in MNI coordinates (min distance between intracluster peaks = 16 mm). Numbers in parentheses in the last column indicate the Brodmann area (BA).

Surprisingly, the stimulus‐locked map showed an overlap with *both* response‐locked maps only at the occipital cortex. No other region with a response‐locked activity profile that was “like‐M1” (e.g., M1, S1, and S2) had an overlap with the stimulus‐locked maps. However, the overlaps of the stimulus‐locked maps with the “unlike‐M1” regions on the response‐locked maps revealed a surprising lateral/medial dissociation. On the lateral cortex, there was an overlap with the peak amplitude map at prePMV (Figure 5b, left panel) that was absent with the peak time map. On the medial cortex, there was an overlap with the peak time map at preSMA (Figure 5b, right panel) that was absent with the peak amplitude map. This was especially notable as large adjacent clusters in the caudal SMA and cingulate gyrus in the peak amplitude map showed no overlap with the stimulus‐locked maps. The prominent clusters in the anterior insula and dorsal anterior cingulate cortex (dACC) on the stimulus‐locked maps did not overlap with either of the response‐locked maps.

Thus, regions that were categorized as having a similar stimulus‐locked activity modulation (i.e., increasing with RT) nevertheless exhibited dissociations when their response‐locked activity profiles were analyzed. Importantly, none of these overlaps, whether at the prePMV or preSMA, were at regions with activity profiles indicating involvement in movement‐generation, that is, like M1. Taken together, these findings are evidence in support of the increased sensitivity predicted from complementing a stimulus‐referenced perspective with a response‐referenced perspective, even with the relatively low time‐resolution of fMRI.

## 4 DISCUSSION

Our strategy here was to use the response protocol in order to read out response‐locked neural effects from the BOLD signal. Multiple lines of evidence suggest that the response protocol achieved this goal. First, we found evidence consistent with an asymmetric sequential relationship between RT and MvT on the hemodynamic state (Figure 3a,b). Second, there was a robust difference in the peak amplitude and peak timing of BOLD activity at the primary motor cortex and somatosensory cortex, consistent with the response‐locked differences between Mov_Short_ and Mov_Long_ in the duration and number of executed movements (Figure 4). There were also strong differences over the occipital cortex consistent with the differences in stimulus display duration between Mov_Short_ and Mov_Long_.

The response protocol itself did not disrupt the stimulus‐related properties of the perceptual decision task. First, the distribution of positive/negative activity modulation with coherence was consistent with prior studies (H R Heekeren et al., 2006; Ho, Brown, & Serences, 2009; Krueger et al., 2017; T. Liu & Pleskac, 2011; Nee & D'Esposito, 2016; Wheeler et al., 2015), especially the study of Heekeren, Marrett, Bandettini, and Ungerleider (2004) that emphasized the role of the dLPFC (Figure 5a). Second, a fundamental property worth emphasizing is that the response protocol enabled the measurement of a *behavioral* RT in the same manner as a traditional response protocol involving a single button press. For instance, in prior studies, a common strategy to isolate pre‐movement information‐processing stages from action execution was to use separate stimuli to provide information about stimulus identity and to command action execution (Filimon et al., 2013; Gold & Shadlen, 2007; Hebart, Donner, & Haynes, 2012; Schouten & Bekker, 1967). Although this “forced” RT strategy (Schouten & Bekker, 1967) provides greater experimental control over the timing of an action, this approach also disrupts the dynamic transformation of the stimulus into an action that response‐locking seeks to access. The availability of behavioral RT with our response protocol provides a common ground truth to integrate findings from fMRI with findings from matched paradigms using high time‐resolution techniques such as EEG and MEG.

We next turn to the question of whether this response protocol revealed any *new* information about the underlying cognitive computations that might otherwise not be available from a stimulus‐locked perspective.

### 4.1 Functional dissociations in perceptual decision‐making

In our paradigm, increasing perceptual difficulty (with decreasing coherence) led to increases in stimulus‐locked activity over the lateral/medial prefrontal cortex (including the anterior cingulate cortex, preSMA, and prePMV) and the anterior insula (Figure 5a). Although several studies have reported this modulation, their underlying computational function to date remains elusive (Filimon et al., 2013; Gold & Shadlen, 2007; Hebart et al., 2012; Ploran et al., 2007; Thura & Cisek, 2014). For instance, it is unclear if they are associated with distinct functions. The response‐locked perspective reveals a dissociation between these regions (Figure 5b).

First, the anterior insula (aIns) and anterior cingulate cortex (ACC) did not show any measurable response‐locked modulations, unlike the preSMA and prePMV. The inclusion of timing revealed a further dissociation. The response‐locked activity at preSMA showed a modulation in timing but not in amplitude while the prePMV showed the opposite pattern. These dissociations implicate differential roles of prePMV, preSMA, and the aIns‐ACC during perceptual decision‐making.

The dissociation between the preSMA and prePMV provides a crucial validation of the value of timing information. For example, with the canonical HRF basis function, the typical activity difference of interest pertains to changes in the amplitude of the hemodynamic response between experimental conditions. Following this tradition, consider the counterfactual scenario where a significant difference in peak amplitude alone was the sole criterion to identify regions showing response‐locked activity differences. Based solely on this criterion, a broad set of regions consistent with Figure 4c (left panel) would show activity differences. Furthermore, the overlap with the coherence‐modulated regions would have solely been at prePMV (Figure 5b, left panel). However, including a timing‐based criterion revealed the existence of an additional overlap with preSMA (Figure 5b, right panel). It might be that the peak time modulation was detected at fewer voxels than the peak amplitude modulation merely due to the low sampling frequency with fMRI (here, TR = 2,200 ms). If this were the case, then the peak time map would be a strict subset of the peak amplitude map. However, this prediction was violated most notably at the preSMA, where there was no measurable difference in peak amplitude.

The properties of the paradigm provide certain constraints for interpreting this putative functional dissociation. When the response is treated as the reference event, it is not solely an output (relative to a stimulus input) but a *transition point* from cognitive choice to movement. It is worth considering the two sides of this transition.

### 4.2 The response‐locked view of perceptual decisions

If a region has a function that operates continuously from the first button press until the end of the trial for both Mov_Short_ and Mov_Long_, then the integrative form of the hemodynamic response function (i.e., a convolution of the HRF with the activation duration) would predict that the BOLD signal has a higher peak amplitude and peak time for Mov_Long_ than for Mov_Short_ (i.e., like M1). This functional demand would be met, for example, by the sensorimotor networks involved in executing individual finger presses and also by the processes involved in monitoring the visual display for the MoveOff stimulus. However, the neural processes triggered by the detection of the MoveOff stimulus (such as processes to inhibit the ongoing movements and reward signals linked to trial completion) only have a brief role at the end of the movement period. Therefore, the corresponding BOLD response evoked by this brief terminating event might have a similar amplitude for Mov_Short_ and Mov_Long_ but with a peak time that is relatively earlier for Mov_Short_ than for Mov_Long_ (Gratton et al., 2017; R. S. Menon et al., 1998; Ploran et al., 2007).

Remarkably, none of the coherence‐modulated regions (except the occipital cortex) had an activity profile modulating both peak amplitude and peak time. Studies on humans and non‐human primates have shown that planning, preparation, and selection of actions can be engaged during perceptual decision‐making (Donner et al., 2009; Filimon et al., 2013; Gold & Shadlen, 2007; H. R. Heekeren et al., 2006; Hauke R. Heekeren et al., 2008; Resulaj et al., 2009; Tosoni et al., 2014; Tosoni et al., 2008). However, the planning of action can occur at many levels of abstraction. One interpretation of movement preparation is that it involves sub‐threshold activation of motor representations that are then activated during movement execution, that is, “raise‐to‐threshold” preparation (Erlhagen & Schöner, 2002; Thura, Beauregard‐Racine, Fradet, & Cisek, 2012; Thura & Cisek, 2014). Such representations might be expected to exhibit activity consistent with the “like M1” template. Contrary to this latter possibility, we found no evidence that motor representations of the kind involved in *executing* an action were active and modulated by RT or stimulus coherence during perceptual decision‐making.

Even though none of the coherence‐modulated regions seemed to have a movement‐related origin, the activity profile at preSMA was consistent with a process triggered by the MoveOff stimulus, namely, a marker of the end of the trial (see above). This result is consistent with the preSMA's previously reported role in context monitoring, sequencing, and task control (Hikosaka & Isoda, 2010; Isoda & Hikosaka, 2007; Nachev, Kennard, & Husain, 2008; Shima & Tanji, 2011) and suggests that the preSMA might have a similar function in the transformation of a stimulus to a response. The preSMA might monitor the start of the response or, possibly, the switch from a perceptually oriented sub‐task (namely, decoding the stimulus) to the demands of action execution.

### 4.3 The stimulus‐locked view of perceptual decisions

In general, to calculate the RT, a “response” marks the earliest time following the stimulus at which the execution of a task‐relevant action can be detected (e.g., a button press. However, this instantaneous binary event is an impoverished description of the actual movement, which takes place over an extended time involving changes in multiple effector‐specific kinematic variables (e.g., velocity, acceleration, forces) (Shenoy, Sahani, & Churchland, 2013; Viswanathan et al., 2019). Typically, these movements are deemed irrelevant to the stimulus‐to‐response transformation for at least two reasons. First, since the response event marks the commitment to a choice, it is assumed that any processes that follow can no longer influence how this choice came to be made (McKinstry, Dale, & Spivey, 2008; but see Spivey, Grosjean, & Knoblich, 2005). Second, a perceptual decision follows from a judgment about a stimulus. Hence, stimulus‐dependent RT changes are assumed to indicate delays in reaching this perceptual decision rather than in executing the subsequent action (Ratcliff & Rouder, 1998).

In the current paradigm, there are two buttons, and each button is associated with a different effector, that is, the left and the right index finger. Therefore, the first press of a button fully specified the choice and further presses of that same button do not provide added information about the choice and cannot change the choice. Many of the processes that are functionally involved in reaching the decision, that is, that *cause* the RT differences to vary with stimulus coherence, or with the immediate consequences of choosing might no longer have a function following the onset of the response.

The anterior insula and the dorsal anterior cingulate cortex have been found to be co‐activated in numerous paradigms and are referred to as being part of a salience network (Medford & Critchley, 2010; V. Menon & Uddin, 2010). The salience network has been hypothesized to be engaged in cognitive control among a variety of functions. Even though the repeated and rapid movements required by the protocol requires cognitive control and appropriate resources, the absence of a modulation of the salience network may suggest that this network has a role in monitoring the *choice* with a limited role after the choice leads to the initiation of the corresponding action.

Along similar lines, the prePMV may indicate the delayed offset of neural activity from the perceptual decision. We hypothesize that prePMV might indicate the continued integration of the decision‐evidence, even though an action has been selected (Rabbitt & Vyas, 1981; Resulaj et al., 2009; Selen, Shadlen, & Wolpert, 2012). The integration continues longer than Mov_Short_ but less than Mov_Long_, so time differences are less evident even though small amplitude differences are present.

### 4.4 An integrated view

**FIGURE 6:**
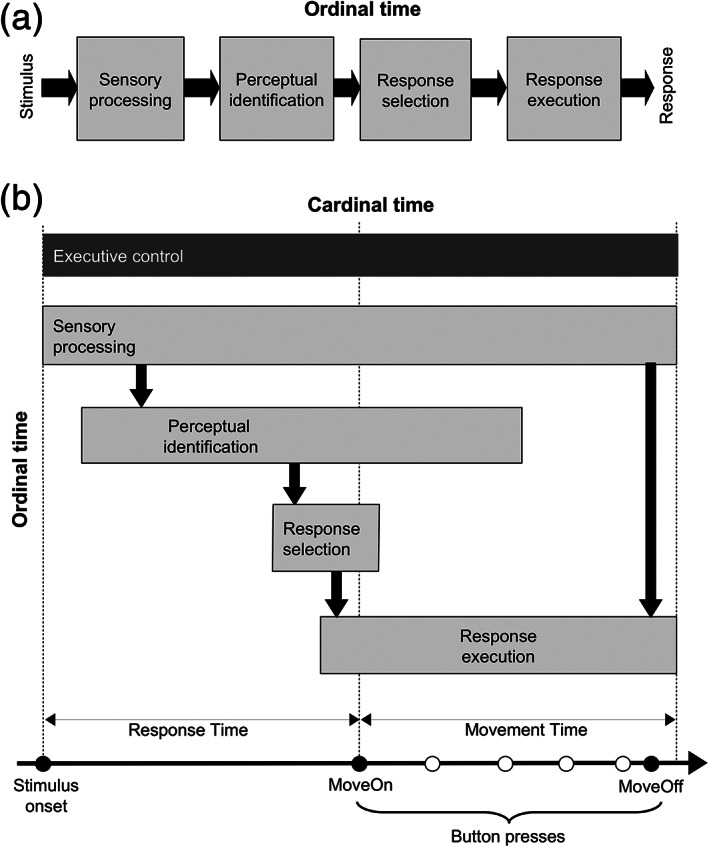
Models. (a) Classical box‐arrow representation of the *ordinal* timeline of cognitive stages to transform a stimulus into a response. (b) Ordinal relationship between stages (stacked vertically) augmented by the *cardinal* timeline (horizontal) for the current paradigm. Thick downward arrows indicate information transfer from stage to stage. The horizontal extension of each box represents the variable *onset* and *offset* of the associated neural processes, which is unspecified in the classical model. The dark gray box (top) indicates the continuous operation of executive control processes over the entire trial

When studying the transformation of a stimulus into action, a traditional representation of the information processing stages is with a series of boxes and arrows, where the arrows represent the ordinal flow of information while the boxes represent hypothetical processors of this information. For instance, a classic model for how perceptual decisions inform action selection is via a processing sequence represented with boxes/arrows: sensory processing, perceptual identification, response selection, and response execution (Figure 6a). These processors perform a transformation, for example, of a stimulus' sensory representation into its identity, or the stimulus identity into an action choice. This is especially crucial for arbitrary stimulus–response mappings where there are two instructed decisions: a decision about the identity of the stimulus (based on an instruction specifying the stimulus features to be evaluated) and a decision about the action to select based on an instructed mapping of stimulus identity to a corresponding response. However, this box‐arrow representation is deceptive about the relative timing of the onset/offset of this processing activity.

When we consider time as a *cardinal* variable, the well‐ordered transition between consecutive stages is difficult to identify due to the unclear onset/offset of activity in brain regions and their roles within these networks. Here we propose an adjustment that allows this box‐arrow representation to be interpreted relative to cardinal time (for a similar formulation obtained by integrating fMRI‐EEG acquired simultaneously see [Muraskin et al., 2018]). In Figure 6b, the sequence of processing stages remains. However, an essential modification is to the onset and offset of the processing‐related stages. Information might pass from stage *X* to the next stage *Y* at a specific time (indicated by the arrows), but the hypothetical “processor” that implements stage *X* might nevertheless continue to be active (i.e., the length of a box along the time dimension). As a consequence, the neural implementations of certain processing stages might seemingly appear over extended periods. Two regions *A* and *B* that are co‐active at a particular time might belong to different functional networks that operate at different times, but the delayed offset of region *A* and the early onset of region *B* might lead to a misattribution that they are involved in performing the same computation. Therefore, a perspective that integrates the stimulus‐locked and response‐locked perspectives is crucial to disentangle not only the onset of a cognitive sub‐process but also its offset.

## 5 CONCLUSION

Despite the comparatively low time‐resolution of fMRI, our findings demonstrate that the choice of reference point has a measurable influence on the sensitivity of measuring timing information with fMRI, much like other high time‐resolution modalities. The novel response protocol introduced here to implement response‐locking involves a minimal change to the typical experimental paradigms that present a stimulus and collect a button press. As demonstrated with a well‐studied perceptual decision‐making task, the increased sensitivity afforded by this small change in response protocol suggests a promising avenue for future research and application to other tasks.

## CONFLICT OF INTEREST

The authors declare no conflicts of interest.

## Supporting information


**Appendix** **S1:** Supporting information

## Data Availability

The data that support the findings of this study are available on request from the corresponding author. The data are not publicly available due to privacy or ethical restrictions.
