# Supplementary material for: A response‐locking protocol to boost sensitivity for fMRI‐based neurochronometry
Source: Hum Brain Mapp. 2020 May 9;41(12):3420–38. doi: 10.1002/hbm.25026 (PMC7375084; doi:10.1002/hbm.25026)
Supplement: Supplementary file 1 — Appendix S1: Supporting information [file HBM-41-3420-s001.docx]

**SUPPLEMENTARY MATERIAL**

**A response-locking protocol to boost sensitivity for**

**fMRI-based neurochronometry**

Shivakumar Viswanathan^1†^, Rouhollah O. Abdollahi^1†^, Bin A. Wang^1^,

Christian Grefkes^1,2^, Gereon R. Fink^1,2^, Silvia Daun^1,3 *^

**TABLE S1: Peak Amplitude (contralateral hemisphere)** Clusters were identified at a threshold of p < 0.05 (FWE, cluster-corrected), cluster threshold = 52 voxels (p<0.0001 uncorr.). Peak locations are in MNI coordinates (min distance between intra-cluster peaks = 16mm). Numbers in parentheses in the last column indicate the Brodmann Area (BA).

| **Cluster Size** | **Peaks** | | | | | | | | | |
| --- | --- | --- | --- | --- | --- | --- | --- | --- | --- | --- |
|  | ***x*** | ***y*** | | ***z*** | | **T value** | | **Anatomical region** | | **Location (BA)** |
| 11923 | -18 | -102 | | -4 | | 15.1 | | Calcarine gyrus | | hOc1 [V1]/hOc2 [V2] (17) |
|  | -26 | -94 | | 12 | | 11.46 | | Middle occipital gyrus | | hOc3d [V3d]/hOc4lp (19) |
|  | -30 | -70 | | -10 | | 9.67 | | Fusiform gyrus | | FG1/hOc4v [V4(v)] (37) |
|  | -26 | -78 | | 20 | | 7.51 | | Middle occipital gyrus | | (19) |
|  | -48 | -70 | | 2 | | 6.91 | | Middle occipital gyrus | | hOc4la/hOc5 [V5/MT] (37) |
|  | -42 | -48 | | -22 | | 6.26 | | Fusiform gyrus | | FG4/FG2 (37) |
| 12695 | -52 | -20 | | 48 | | 13.28 | | Postcentral gyrus | | 1/3b (1) |
|  | -36 | -24 | | 60 | | 12.84 | | Precentral gyrus | | **M1** (4a, 4) |
|  | -50 | -32 | | 60 | | 11.88 | | Postcentral gyrus | | SI (1) |
|  | -16 | -20 | | 6 | | 10.13 | | Thalamus | | Thal: Premotor/Prefrontal |
|  | -46 | -22 | | 18 | | 9.68 | | Frontal operculum | | OP1 [SII]/OP3 [VS] (41) |
|  | -40 | -4 | | 12 | | 9.01 | | Insula lobe | | OP3 |
|  | -24 | 0 | | 4 | | 8.63 | | Putamen | |  |
|  | -52 | 2 | | 34 | | 8.61 | | Precentral gyrus | | **vPMC** (44, 6) |
|  | -2 | -4 | | 74 | | 7.74 | | Dorsal medial frontal cortex | | **SMA/preSMA** (6) |
|  | -48 | -36 | | 26 | | 7.48 | | Supramarginal gyrus | | PFcm (41) |
|  | -8 | 6 | | 36 | | 7.42 | | Middle cingulate cortex | | aMCCd (24) |
|  | -2 | -12 | | 54 | | 7.02 | | Dorsal medial frontal cortex | | **SMA** (6) |
|  | -34 | -54 | | 52 | | 6.56 | | Superior parietal lobule | | SPL (7A)/hIP3, IPS (7) |
|  | -52 | 18 | | 26 | | 6.33 | | IFG (pars triangularis) | | **prePMV (**44/45) |
|  | -56 | | 8 | | 12 | | 6.07 | | IFG (pars opercularis) | **vPMC** (44, 6) |
|  | -18 | | -6 | | 18 | | 5.93 | | Thalamus | Thal: Temporal/Parietal |
|  | -18 | | -64 | | 44 | | 5.35 | | Superior parietal lobule | SPL (7A/7) |
| 66 | -40 | | 34 | | 16 | | 5.41 | | IFG (pars triangularis) | (45/46) |
| 52 | -38 | | 22 | | 0 | | 5.1 | | Insula lobe | Ialn |

**TABLE S2: Peak Time (contralateral hemisphere)** Clusters were identified at a threshold of p < 0.05 (FWE, cluster-corrected), cluster threshold = 41 voxels (p<0.0001 uncorr.). Peak locations are in MNI coordinates (min distance between intra-cluster peaks = 16mm). Numbers in parentheses in the last column indicate the Brodmann Area (BA).

| **Cluster Size.** | **Peaks** | | | | | |  |
| --- | --- | --- | --- | --- | --- | --- | --- |
|  | ***x*** | ***y*** | ***z*** | **T value** | **Anatomical region** | **Location (BA)** | |
| 9019 | -24 | -90 | -6 | 16.58 | Inferior occipital gyrus | hOc3v [V3v] (19) | |
|  | -12 | -98 | 2 | 13.14 | Middle occipital gyrus | hOc1 [V1] (17) | |
|  | -32 | -72 | -10 | 11.33 | Fusiform gyrus | FG1/hOc4v [V4(v)] (37) | |
|  | -34 | -86 | 8 | 8.98 | Middle occipital gyrus | hOc4lp/hOc4la (19) | |
|  | -44 | -64 | 0 | 8.81 | Middle temporal gyrus | hOc5 [V5/MT] (37) | |
|  | -24 | -96 | 20 | 7.67 | Superior occipital gyrus | hOc3d [V3d]/hOc4d [V3A] (19) | |
|  | -36 | -56 | -20 | 7.32 | Fusiform gyrus | FG2 (37) | |
|  | -18 | -66 | -18 | 6.48 | Cerebellum (VI) | Lobule VI (Hem) | |
|  | -28 | -78 | 22 | 5.58 | Middle occipital gyrus | (19) | |
| 3486 | -40 | -18 | 50 | 16.04 | Precentral gyrus | **M1 (**4a/4p, 3) | |
|  | -34 | -24 | 68 | 11.78 | Precentral gyrus | **M1 (**4) | |
|  | -50 | -16 | 18 | 9.83 | Postcentral gyrus | OP3 [VS]/OP4 [PV] (41) | |
|  | -42 | -34 | 48 | 7.13 | Postcentral gyrus | 2/3b (2) | |
|  | -60 | -38 | 22 | 6.44 | Superior temporal gyrus | PF (IPL)/PFcm (IPL) (42) | |
|  | -28 | -4 | 60 | 4.95 | Precentral gyrus | **dPMC** (6) | |
|  | -34 | -24 | 18 | 4.88 | Insula lobe | OP2 [PIVC]/Ig1 (OP2) | |
|  | -58 | -28 | 38 | 4.65 | Supramarginal gyrus | PFt (IPL)/PF (IPL) (40) | |
| 832 | -6 | -2 | 56 | 11.5 | Dorsal medial frontal cortex | **SMA/preSMA** (6) | |
| 390 | -12 | -16 | 8 | 9.71 | Thalamus | Thal: Prefrontal/ Temporal | |
|  | -12 | -18 | -8 | 5.35 | Thalamus |  | |
| 351 | -56 | 2 | 2 | 8.15 | Frontal operculum | TE 3/TE 1.2 | |
|  | -58 | 10 | 28 | 6.85 | Precentral gyrus | **vPMC** (44/45, 6) | |
| 64 | -12 | -70 | -38 | 6.13 | Cerebellum (VIII) | (19) | |
| 41 | -46 | 2 | 40 | 6.03 | Precentral gyrus | **dorsal** **vPMC (**44, 6) | |
| 56 | -28 | -52 | 50 | 5.2 | Superior parietal lobule | hIP3/IPS/SPL (7) | |
